# Golden Gate Cloning-Compatible DNA Replicon/2A-Mediated Polycistronic Vectors for Plants

**DOI:** 10.3389/fpls.2020.559365

**Published:** 2020-10-21

**Authors:** Jae Hoon Lee, Hyo Jun Won, Eun-Seok Oh, Man-Ho Oh, Je Hyeong Jung

**Affiliations:** ^1^Smart Farm Research Center, Korea Institute of Science and Technology, Gangneung, South Korea; ^2^Division of Bio-Medical Science & Technology, KIST School, Korea University of Science and Technology, Daejeon, South Korea; ^3^Department of Biological Sciences, College of Biological Sciences and Biotechnology, Chungnam National University, Daejeon, South Korea

**Keywords:** Golden Gate cloning, Replicon, 2A peptides, polycistronic expression, plant metabolic engineering

## Abstract

The expression of multiple proteins and high-throughput vector assembly system are highly relevant in the field of plant genetic engineering and synthetic biology. Deployment of the self-cleaving 2A peptide that mediates polycistronic gene expression has been an effective strategy for multigene expression, as it minimizes issues in coordinated transgene regulation and trait staking in plants. However, efficient vector assembly systems optimized for 2A peptide-mediated polycistronic expression are currently unavailable. Furthermore, it is unclear whether protein expression levels are influenced by the transgene position in the polycistronic expression cassette. In this article, we present Golden Gate cloning-compatible modular systems allowing rapid and flexible construction of polycistronic expression vectors applicable for plants. The genetic modules comprised 2A peptides (T2A and P2A)-linked tricistron expression cassette and its acceptor backbones, named pGO-DV1 and pGO-DV2. While both acceptor backbones were binary T-DNA vectors, pGO-DV2 was specially designed to function as a DNA replicon enhancing gene expression levels. Using the Golden Gate cloning, a set of six tricistronic vectors was constructed, whereby three transgenes encoding fluorescent proteins (mCherry, eYFP, and eGFP) were combinatorially placed along the expression cassette in each of the binary vectors. Transient expression of the construct in tobacco leaves revealed that the expression levels of three fluorescent proteins were comparable each other regardless of the gene positions in the tricistronic expression cassette. pGO-DV2-based constructs were able to increase protein expression level by up to 71%, as compared to pGO-DV1-based constructs.

## Introduction

There has been a pressing need to accelerate the pace of crop improvement recently, in order to meet the challenges presented by the growing population and rapid climate change. Although conventional breeding approaches have considerably improved crop productivity and end-use quality over the past century, they present several limitations as the probability of success is largely dependent on the genetic variability and architecture of target traits (Schaart et al., 2016). Thereupon, extensive efforts have been focused on enhancing biotechnological approaches for directly manipulating genetic and metabolic pathways in plants.

Successful multiple-gene integration is an attractive prospect for the genetic improvement of plants, especially when attempting to accumulate desired traits of interest into elite cultivars, systemically manipulate and optimize metabolic networks, or boost biochemical production via metabolic engineering. In plants, the integration of multiple genes has been pursued through sequential crossing between pre-existing independent transgenic lines, and by introducing multiple expression cassettes through a single co-transformation process. However, these strategies often inhibit the generation of elite transgenic lines due to complex transgene segregation patterns among the lines and largely variable transgene expression levels, depending on the transgene integration loci in the genome (Vaucheret et al., 1998; Lechtenberg et al., 2003; de Felipe et al., 2006). While the introduction of a single vector containing multiple expression cassettes could be an option, successful synchronized gene expression would be limited as each expression cassette would have to be under the control of different regulatory elements in order to avoid the occurrence of co-suppression events (Fagard and Vaucheret, 2000; Daniell and Dhingra, 2002). Owing to these limitations, polycistronic expression derived from a single expression cassette is regarded as an alternative strategy which would allow stable co-segregation, uniform gene expression, and synchronized regulation of multiple transgenes (Meyers et al., 2010; Cermak et al., 2017).

Polycistronic expression is mediated by linker sequences in the form of either RNA or peptides. A viral peptide consisting of 18–22 amino acids, known as 2A peptide, has been widely used as a self-cleaving linker sequence in the polycistronic expression system. The first discovered 2A peptide was F2A from the foot-and-mouth disease virus; others, such as E2A (equine rhinitis A virus), P2A (porcine teschovirus), and T2A (thosea asigna virus), were subsequently identified (Ryan et al., 1991; Donnelly et al., 2001a). All members of the 2A peptide class contain the DxExNPGP consensus motif at the C-terminus, with the cleavage site located between the last two amino acids, glycine (G) and proline (P) (Donnelly et al., 2001b; de Felipe et al., 2003). The current model for the mode of action proposed that 2A peptide induces translation termination via skipping a peptide bond formation in the ribosome, and allows continued translation of the downstream product (Atkins et al., 2007; Doronina et al., 2008). Since the cleavage is mediated by ribosomes rather than proteases or other cellular factors, the 2A-mediated polycistronic expression system has been efficient in a wide range of eukaryotic organisms, including plants (Halpin et al., 1999; de Felipe et al., 2003; Szymczak et al., 2004). For example, in maize, 11 different genes which were successfully introduced using a 2A-linked polycistronic construct remained stacked together in the genome, stably inherited, until the T4 generation (Liu et al., 2018). Therefore, in the field of plant genetic and metabolic engineering, the use of 2A-linked polycistronic vectors could not only be a promising strategy for multiple protein expression, but also a viable alternative for minimizing related issues with the integration of multiple genes (Halpin, 2005).

Vector construction is a fundamental step in a wide range of genetic and metabolic engineering processes. Golden Gate cloning is one of the most efficient methods for vector construction (Engler et al., 2008; Weber et al., 2011). This approach is based on the unique ability of Type IIS endonuclease to cleave outside of its recognition site, leaving customizable overhang sequences as potential ligation sites. Genetic elements modularized to carry compatible ligation sites and inward-facing recognition sites for Type IIS endonuclease can be assembled with simultaneous digestion and ligation reactions in a single tube. Compared to traditional cloning strategies, Golden Gate cloning allows rapid and precise combinatorial assembly of multiple DNA fragments.

In spite of the applicability of polycistronic systems for integrating multiple genes in plants, limited genetic resources and cloning methods are available for the construction of 2A peptide-linked polycistronic vectors, a process still reliant either on time- and cost-consuming Type IIP restriction enzyme-based methods, or on recombination events following the addition of overlapping flaking sequences (Ha et al., 2010; Lee et al., 2012; Liu et al., 2018). In addition, it is unclear whether protein expression levels are influenced by the transgene position in the polycistronic expression cassette. In an attempt to efficiently exploit 2A-mediated polycistronic expression systems in plants, the present study aimed to establish a convenient and flexible cloning procedure for vector construction and to verify the relationship between the expression levels and relative gene positions in the polycistronic cassette. We applied synthetic biology approaches and constructed a fundamental set of Golden Gate cloning-compatible modular vectors which comprised the expression cassette and acceptor backbones, named pGO-DV1 and pGO-DV2. Both acceptor backbones were binary T-DNA vectors suitable for plant transformation. pGO-DV2 was specially designed to possess the geminiviral replicon system, capable of producing circular DNA replicons for high-level multiple protein expression. Using the Golden Gate cloning approach, a set of six 2A-linked tricistronic vectors was constructed, whereby three transgenes encoding fluorescent proteins (FPs) (mCherry, eYFP, and eGFP) were combinatorially placed along the expression cassette in each of the binary vectors. This study investigated expression levels of the polycistron and its encoded proteins within the vector combination thorough transient expression in tobacco leaves. Functional protein expression from the polycistron was also confirmed *in planta* using confocal microscopy.

## Materials and Methods

### Golden Gate-Compatible Modular Vector Construction

**FIGURE 1:**
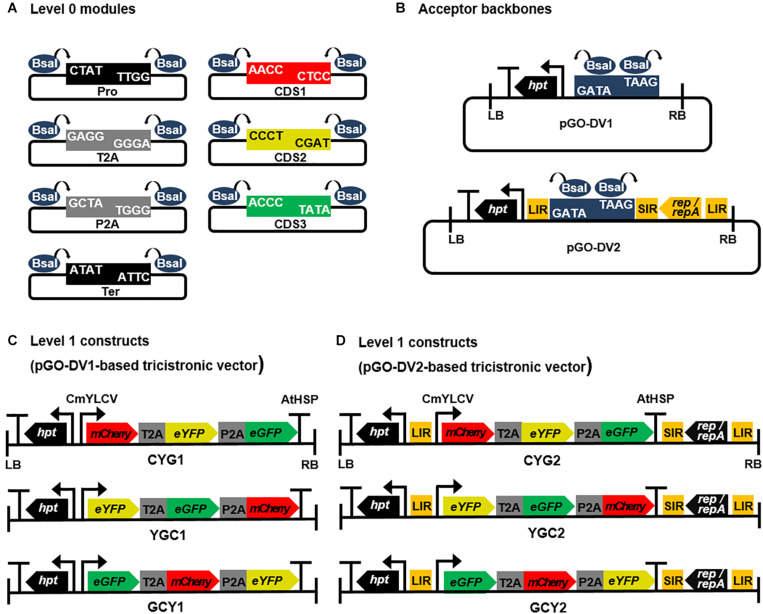
Schematic diagrams of Golden Gate cloning-compatible modular vectors and assembled 2A peptide-linked tricistronic expression cassettes. **(A)** A set of Golden Gate cloning-compatible level 0 modular vectors. All modules were flanked by inward-facing two *Bsa*I restriction enzyme sites and customized 4 bp-overhangs to allow directional linear assembly in a Pro-CDS1-T2A-CDS2-P2A-CDS3-Ter orientation. Pro, promoter; CDS1, CDS2, and CDS3, coding sequences; T2A and P2A, T2A and P2A peptide coding sequences; Ter, terminator. **(B)** level 1 acceptor backbone modules containing *Bsa*I-mediated Golden Gate cloning site. Both acceptor backbones were constructed based on the pLSLR vector (Baltes et al., 2014), and they were binary T-DNA vectors containing hygromycin selection marker (*hpt* gene) and left/right borders (LB/RB). In pGO-DV1, *Bsa*I-mediated Golden Gate cloning site was inserted by removing *cis*-acting replicational elements (LIR, SIR, and LIR) and genes encoding replication proteins (Rep/RepA), whereas the cloning site was inserted in between LIR and SIR to retain replicon producing function in pGO-DV2. **(C,D)** The assembled T2A and P2A-linked tricistronic expression cassettes harboring three transgenes encoding fluorescent proteins (mCherry, eYFP, and eGFP). The genes were combinatorially placed along the expression cassette in each of the acceptor backbone vectors, and each of the construct was denoted by CYG1, YGC1, GCY1, CYG2, YGC2, or GCY2. The promoter and terminator in the constructs were CmYLCV and AtHSP, respectively.

Using the assembly procedure described in the Golden Gate modular cloning system (Weber et al., 2011), “level 0” modular vectors containing parts of the expression cassette, such as promoter (Pro), 2A signals (T2A and P2A), coding sequences (CDS), and terminator (Ter), were constructed (Figure 1A). All level 0 modules were flanked by inward-facing *Bsa*I restriction enzyme sites and fusion sites (4 bp-overhangs) to allow directional linear assembly in a Pro-CDS1-T2A-CDS2-P2A-CDS3-Ter orientation, resulting in 2A-linked tricistronic constructs. In order to construct Pro, 2A, and Ter modules, sequences of CmYLCV promoter, 2A signals (T2A and P2A), and AtHSP 3′ UTR were retrieved from publications by Stavolone et al. (2003), Nagaya et al. (2010), and Liu et al. (2017), respectively. T2A and P2A sequences were codon-optimized for *Nicotiana tabacum* using the Codon Usage Database^1^. Pro, 2A, and Ter modules were custom-synthesized at Bioneer (Daejeon, South Korea), and harbored into the pLUG-Prime vector (iNtRON Biotechnology, South Korea). CDS modules encoding FPs (mCherry, eYFP, and eGFP) were prepared without codon-optimization by PCR using primers carrying inward-facing *Bsa*I sites and fusion sites as listed in Supplementary Table 1. DNA templates for each CDS were gifts from Moon-Hyeong Seo (KIST, Gangneung, South Korea). To investigate the relative expression levels at different transgene positions in a tricistronic construct, a set of three CDS modules for each FP (i.e., a total of nine modules) was prepared and assembled into the CDS1, CDS2, or CDS3 position.

“Level 1” acceptor backbones were constructed based on the pLSLR vector (Baltes et al., 2014). The vector was a gift from Daniel Voytas (Addgene plasmid # 51500^2^; RRID:Addgene_51500). Firstly, CaMV 35S promoter flanked by HSP70 intron was inserted into *Avr*II and *Sal*I-digested pLSLR, and the resulting vector was named pLSLR-35SHSP (Supplementary Figure 1A). A fragment flanked by two *Bsa*I sites (5′-CTATGGAGACCGAGGTCTCGTAAG-3′) for Golden Gate cloning was then inserted into pLSLR-35SHSP at two different sites: cloning into *Sal*I- and *Pme*I-digested pLSLR-35SHSP formed pGO-DV1 (Supplementary Figure 1B and Figure 1B), while cloning into *Pml*I- and *Xho*I-digested pLSLR-35SHSP formed pGO-DV2 (Supplementary Figure 1C and Figure 1B). Vector maps and full sequence information of “level 0” modules and “level 1” acceptor backbones are available through the Addgene repository (Addgene ID 160370, 160371, 160372, 160373, 160374, and 160375).

To construct 2A-linked tricistronic vectors in a Pro-CDS1-T2A-CDS2-P2A-CDS3-Ter orientation, level 0 modules were directionally assembled into the level 1 acceptor backbone using a single digestion-ligation procedure. The T2A-P2A order was chosen due to its higher cleavage efficiency and protein expression compared to other 2A combinations based on previous studies (Kim et al., 2011; Liu et al., 2017). An equal molar ratio of level 0 modules and level 1 acceptor was mixed with *Bsa*I (NEB, Ipswich, MA, United States) and T4 ligase (NEB). The reaction was carried out for 10 cycles of 5 min at 37°C and 10 min at 16°C, followed by 5 min at 50°C and 5 min at 80°C. Assembled level 1 constructs were amplified in *Escherichia coli* DH5α, and the subsequent plasmid recovery, restriction digestion, and sequencing procedures confirmed correct vector assembly. A total of six tricistronic vectors were constructed to possess different relative positions of each CDS and acceptor backbones (Figures 1C,D). Each tricistronic vector was transformed into *Agrobacterium tumefaciens* LBA4404 by electroporation for further studies.

### Transient Expression of Fluorescent Proteins in Tobacco Leaves

*Nicotiana tabacum* cv. Samsun were grown in a growth chamber under 24/21°C (day/night), 16 h photoperiod, 500 μmol/m^2^/s light intensity, and 80% relative humidity. *Agrobacterium* cells carrying the indicated 2A polycistronic construct were grown in LB medium, harvested, and adjusted to OD_600_ 0.4 in resuspension solution (10 mM MgCl_2_, 10 mM MES-KOH pH 5.6, 100 μM acetosyringone). After incubation for 3 h at room temperature, bacterial suspension was infiltrated into 4- to 5-week-old *N. tabacum* leaves using needless syringe and incubated in a growth chamber with the same conditions described above. Leaves were collected at 3 and 5 days post-infiltration (DPI) for the quantification of RNA and protein expression levels.

### RNA Isolation and Quantitative Real-Time PCR

Total RNA was isolated from plant leaves using TRIzol (Life Technologies, Carlsbad, CA, United States) and RNeasy Plant RNA kit (Qiagen, Hilden, Germany), followed by DNase treatment and reverse transcription with Ambion DNA-free kit (Thermo Fisher Scientific, Vilnius, Lithuania) and high capacity cDNA reverse transcription kit (Thermo Fisher Scientific), respectively, according to the manufacturer’s instruction. Quantitative real-time PCR (qRT-PCR) was performed using 2X real-time PCR SYBR green master mix (Biofact, Daejoen, South Korea) on Applied Biosystems 7500 real-time PCR system (Applied Biosystems, Foster City, CA, United States) under the following conditions: 95°C for 10 min, followed by 40 cycles of 95°C for 15 s and 60°C for 1 min. The *L25* ribosomal protein gene was used as an internal control for normalization. Data are mean of three biological replicates, each from an independent experiment, and two technical replicates were performed for each biological replicate. Primers used in qRT-PCR analysis are listed in Supplementary Table 1.

### Plant Protein Extraction and Quantification of Fluorescent Proteins

Soluble proteins from plant leaves were extracted using the P-PER plant protein extraction kit (Thermo Fisher Scientific) according to the manufacturer’s instructions, and their protein concentration was determined by Bradford assay. Fluorescence intensity of the extracted proteins was measured using a Tecan Infinite M1000Pro microplate reader (Tecan, Männedorf, Switzerland). The excitation/emission spectra were 488/507 nm for eGFP, 514/527 nm for eYFP, and 587/610 nm for mCherry. Each fluorescence protein amount was determined using standard curves generated from serial dilutions of His-tagged recombinant FPs (Supplementary Figure 2), which were purified as previously described (Lee et al., 2019). Quantification was performed after dilution of leaf soluble proteins so that fluorescence signals fell within the linear range of the standard curve. Data are mean of three biological replicates, each from an independent experiment, and two technical replicates were performed for each biological replicate. Primers used in the cloning of recombinant proteins are listed in Supplementary Table 1.

### Imaging of Fluorescent Proteins in Tobacco Leaves

Fluorescent protein expression in tobacco leaves were observed at 5 DPI under a LSM5 Zeiss confocal laser scanning microscope using ZEN image analysis software (Carl Zeiss, Jena, Germany). The excitation/emission spectra were 488 nm/493 to 598 nm for eGFP, 514 nm/517 to 588 nm for eYFP, and 594 nm/599–650 nm for mCherry. Detector gain setting was adjusted due to fluorescent intensity differences between pGO-DV1- and pGO-DV2-based tricistronic constructs: fluorescence from pGO- DV1 constructs was imaged at 505 for eGFP, 615 eYFP, and 760 for mCherry, while that from pGO-DV2 constructs was imaged at 400 for eGFP, 500 for eYFP, and 520 for mCherry. Presented data is representative of three biological replicates.

### Statistical Analysis

Using R, analysis of variance (ANOVA) with Tukey’s honestly significant differences (HSD) tests were performed to determine statistical differences among means of each FP abundance within the construct. *t-*tests were also performed to determine statistical mean differences between two independent groups, such as a comparison of means of polycistron abundance and total FPs between pGO-DV1- and pGO-DV2-based constructs at each DPI.

## Results and Discussion

DNA assembly and cloning methodologies have greatly improved following the concept of synthetic biology (Ellis et al., 2011). The modularization of genetic elements followed by Golden Gate cloning has been a key technology in enabling high-throughput DNA assembly and facilitating the so called “Design-Build-Test cycle” in order to evaluate new genetic circuits, and subsequently advanced our understanding of biological systems (Weber et al., 2011). Modularized genetic elements also serve as potential resources for genetic and metabolic engineering. While libraries of various genetic modules including promoters, terminators, localization signals, and selectable markers have been available for biotechnological applications (Sarrion-Perdigones et al., 2013; Engler et al., 2014), their implementations in polycistronic expression systems have not been reported thus far.

The use of 2A self-cleaving peptide for polycistronic expression requires the addition of the 2A encoding DNA fragment (∼60 bp) between genes of interest, in the form of a single open reading frame (ORF). Traditional cloning methods, which rely on Type IIP endonucleases, present difficulties in selecting proper restriction sites to ligate 2A and CDS without disrupting the ORF. Furthermore, it is a laborious and time-consuming process as enzyme digestion, followed by purification and ligation, has to be performed separately. In the present study, we report for the first time the construction of robust and efficient modular vectors which enable a high-throughput assembly of 2A-linked polycistronic vectors for plants. As shown schematically in Figure 1, a total of seven modules were precisely assembled in a predefined order to form a tricistronic expression cassette using simultaneous enzyme digestion and ligation reactions. In addition, the Golden Gate cloning process took only 3 days, including a 2-day period of bacterial transformation and culture. The majority of the bacterial clones (∼90%) contained the correctly assembled plasmid.

Together with a convenient vector assembly process, the ability to boost gene expression levels is an important feature in vector system designed for biotechnological applications. Geminiviral vectors are capable of producing DNA replicons inside host cells, and thus have been used for achieving high-level expression of heterologous proteins in plants (Mor et al., 2003; Huang et al., 2010; Regnard et al., 2010; Baltes et al., 2014; Diamos and Mason, 2019). They carry *cis*-acting replicational elements (LIR-SIR-LIR; LSL) and genes encoding replication proteins (Rep/RepA), which allow the rolling-circle replication of transgenes. As one of the geminiviral vectors viable for plant transformation, pLSLR derived from bean yellow dwarf virus (BeYDV) was engineered for the Golden Gate-compatible vector construction. A previous study has determined that this vector can be successfully employed for genome editing, based on its ability to increase the expression levels of editing agents (Baltes et al., 2014). To date, no attempt has been made to apply the DNA replicon system in 2A-mediated polycistronic expression. During the process of vector construction, the replicon producing function was removed from pGO-DV1 and retained in pGO-DV2, in order to compare expression levels between the two (Figure 1B).

The use of FPs is a straightforward approach to visualize functional protein expression and to quantify their expression levels. Here, we utilized three FPs (mCherry, eYFP, and eGFP; hereafter denoted by C, Y, and G, respectively) to evaluate the efficacy and feasibility of the modular vector systems, especially for tricistronic expression. As shown in Figures 1C,D, we generated six different tricistronic vectors with CYG, YGC, or GCY orientation, in either the pGO-DV1 (CYG1, YGC1, GCY1) or pGO-DV2 (CYG2, YGC2, GCY2) acceptor backbone. T2A and P2A peptides were inserted in the first and second intergenic regions, respectively, in all the vectors.

**FIGURE 2:**
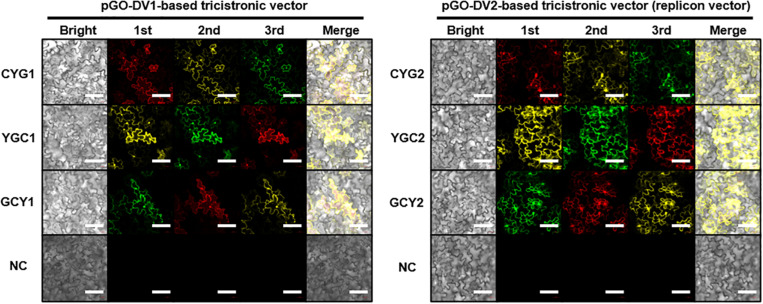
Confocal microscope images of fluorescent proteins at 5 days after agroinfiltration of tricistronic constructs into tobacco leaves. Images in the red channel (excitation: 594 nm; emission: 599–650 nm), yellow channel (excitation: 514 nm; emission: 515–588 nm), and green channel (excitation: 488 nm; emission 493–598 nm) represent mCherry (C), eYFP (Y), and eGFP (G), respectively. Resuspension solution buffer was used as the negative control. Presented data is representative of three biological replicates. Bright, bright-field image; 1st, 2nd, and 3rd, images of fluorescent protein at each position within the tricistronic construct; merge, merge of bright-field and fluorescence images; NC, negative control. White bar indicates 100 μm.

**FIGURE 3:**
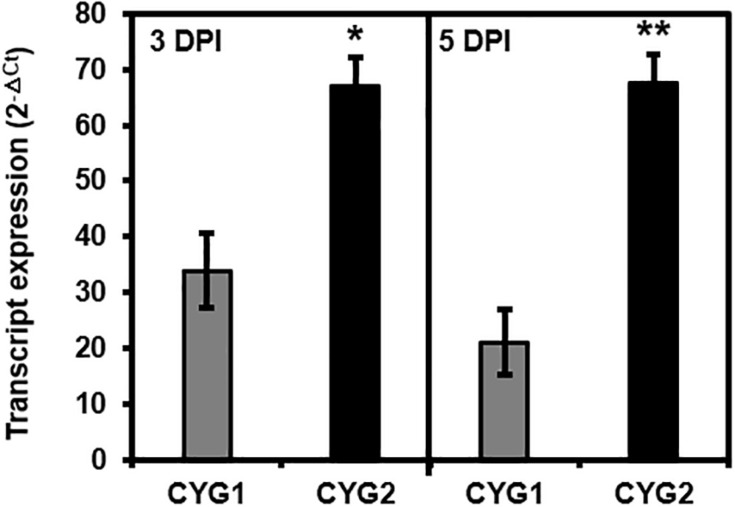
Tricistron expression levels from CYG1 and CYG2 constructs at 3 and 5 days after agroinfiltration into tobacco leaves. Expression levels were determined as 2^–ΔCt^ using the *L25* gene as an endogenous control, and data represent the mean value of three biological replicates, each with two technical replicates. Statistical significance of differences between CYG1 and CYG2 at each day was assessed by *t*-test with probability values of **p < 0.001 and *p < 0.01. Error bars indicate standard deviation. DPI, days post-infiltration; Ct, cycle threshold.

Following the agroinfiltration of individual tricistronic constructs into tobacco leaves, functional protein expression was confirmed using confocal microscopy imaging (Figure 2). At 5 DPI, pGO-DV2-based tricistronic vectors exhibited a 1.7- to 2.3-fold increase in fluorescence intensity (calculated using the arithmetic mean) compared to pGO-DV1-based tricistronic vectors, indicating that the pGO-DV2 acceptor backbone replicated transgene copies as intended. The pGO-DV2-mediated increment of transgene expression was also verified at the transcription level (Figure 3). Tricistron expression levels from CYG2 were 2.0- and 3.2-fold higher at 3 and 5 DPI, respectively, compared to levels from CYG1.

**FIGURE 4:**
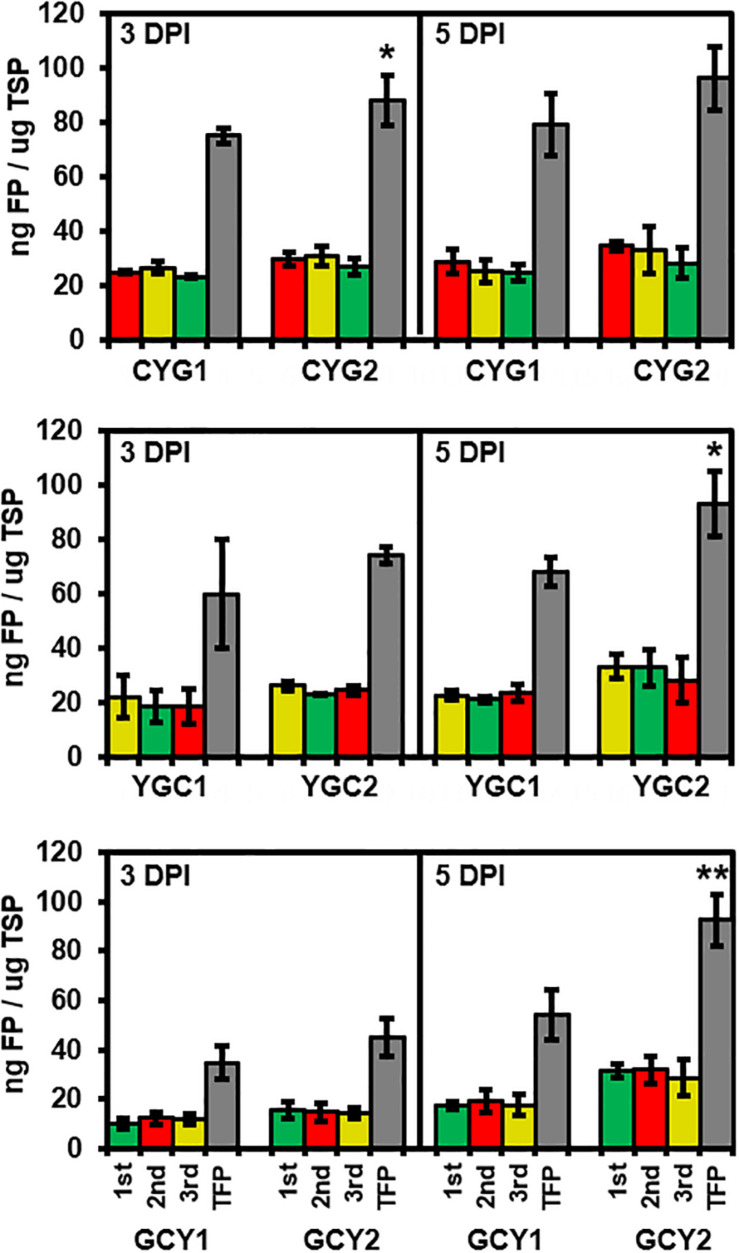
Evaluation of tricistronic constructs for protein abundance and positional effects at 3 and 5 days after agroinfiltration into tobacco leaves. Data represent the mean values of fraction of each fluorescent protein (mCherry, red column; eYFP, yellow column; eGFP, green column) or total fluorescent protein (TFP, gray column) to total soluble proteins (TSP), obtained from three biological replicates, each with two technical replicates. Abundance of each fluorescent was quantified using standard curves relating fluorescence intensity to protein concentration, and TSP concentration was quantified using Bradford assay. Statistical significance of differences between TFPs from pGO-DV1- and pGO-DV2-based constructs at each day was assessed by *t*-test with probability values of **p < 0.01 and *p < 0.05. No statistical difference (*p* < 0.05) was detected between abundances of each FP within the constructs, according to the analysis of variance (ANOVA) and Tukey’s honestly significant differences (HSD) tests. Error bars indicate standard deviation. DPI, days post-infiltration.

Expression levels of each FP were quantified from all six tricistronic constructs (Figure 4). Total soluble proteins (TSP) were isolated at 3 and 5 DPI from an area of tobacco leaves subjected to agroinfiltration, and the amount of each FP was quantified based on the standard curve for the respective FP. In all collected samples, the amounts of each FP translated from the same tricistronic construct were not significantly different, based on the ANOVA with *post hoc* Tukey HSD test (red, yellow, and green bars in Figure 4). The average amount of individual FPs produced from the tricistronic vector system ranged from 12 to 32 ng FP/μg TSP at 3 and 5 DPI, while GCY1 constructs tended to produce a lower amount of FP compared to either CYG1 or YGC1. The total amount of FP (TFP; sum of the amount of each FP) was also compared between pGO-DV1- and pGO-DV2-based tricistronic constructs. Similar to the result obtained from microscopic and transcript analyses, pGO-DV2-based tricistronic constructs showed 17–71% more TFP accumulation than pGO-DV1-based constructs at 3 and 5 DPI (Gray bars in Figure 4). In particular, almost 10% of the TSP accounted for TFP at 5 DPI of pGO-DV2-based tricistronic constructs. CYG2, YGC2, and GCY2 produced 96, 93, and 93 ng TFP/μg TSP, respectively, where TFP was increased by 22, 27, and 71%, respectively, compared to values from corresponding pGO-DV1-based vectors. In addition, western blot was also performed on TSP of GCY1 and GCY2 using GFP antibody, and the majority of detected bands had an approximate molecular weight of about 27 kDa, indicating actual cleavage of fusion proteins *in planta* (Supplementary Figure 3).

In theory, the 2A-mediated polycistronic system allows for the production of multiple proteins at equimolar levels, as translation is carried out from a single polycistron, and peptides linked by 2A peptide are separated via a ribosome skipping mechanism. However, it should be noted that expression levels from 2A polycistronic constructs are dependent on the combined effects of various factors, including the number of transgenes, their relative positions in the construct, the order and types of 2A peptide, 2A coding sequences, as well as regulatory elements in the expression cassette (Donnelly et al., 2001a; de Felipe et al., 2010; Rothwell et al., 2010; Gao et al., 2012; Liu et al., 2017). With regards to the effects of transgene position, the results of this study are similar to those of a previous report which showed that no significant differences in transgene expression levels were observed when varying the positions within 2A polycistronic constructs in mouse embryonic stem cells (Gao et al., 2012). On the other hand, transgenes located distant to the first position tended to display reduced translation levels in mouse cell lines, while transgene activities were lowest at the first position compared to others in *Aspergillus niger* (Liu et al., 2017; Schuetze and Meyer, 2017). From these collective observations, an empirical study would be preferred for predicting protein expression levels in newly generated 2A-linked polycistronic constructs. In this respect, the Golden Gate cloning system presented here could be highly beneficial for the rapid construction of new vectors in a combinatorial manner, and, following transient expression analysis using report systems, could further facilitate the selection of optimal assembly combinations for biotechnological applications in plants.

## Conclusion

In summary, our new Golden Gate cloning-compatible modules allows the rapid and flexible construction of 2A-linked polycistronic vectors for plants. Under the given module combination in a Pro-CDS1-T2A-CDS2-P2A-CDS3-Ter orientation, comparable transgene expression can be expected regardless of the gene positions in the tricistronic expression cassette. Furthermore, expression levels of the polycistron and its encoded proteins can be increased by utilizing the replicon vector system, pGO-DV2. The vector systems presented in this study can also be applicable for stable transformation, as well as serve as novel genetic resources for broadening the choice of vectors for plant genetic and metabolic engineering.

## Data Availability Statement

The raw data supporting the conclusions of this article will be made available by the authors, without undue reservation.

## Author Contributions

JL collected and analyzed the data and wrote the manuscript. HW, E-SO, and M-HO collected the data. JJ designed the study, analyzed the data, and wrote the manuscript. All authors revised and approved the manuscript.

## Conflict of Interest

The authors declare that the research was conducted in the absence of any commercial or financial relationships that could be construed as a potential conflict of interest.

## Footnotes

1 http://www.kazusa.or.jp/codon/

2 http://n2t.net/addgene:51500
