# Supplementary material for: Golden Gate Cloning-Compatible DNA Replicon/2A-Mediated Polycistronic Vectors for Plants
Source: Front Plant Sci. 2020 Oct 21;11:559365. doi: 10.3389/fpls.2020.559365 (PMC7609577; doi:10.3389/fpls.2020.559365)
Supplement: Supplementary file 1 [file Presentation_1.PPTX]

## Slide 1
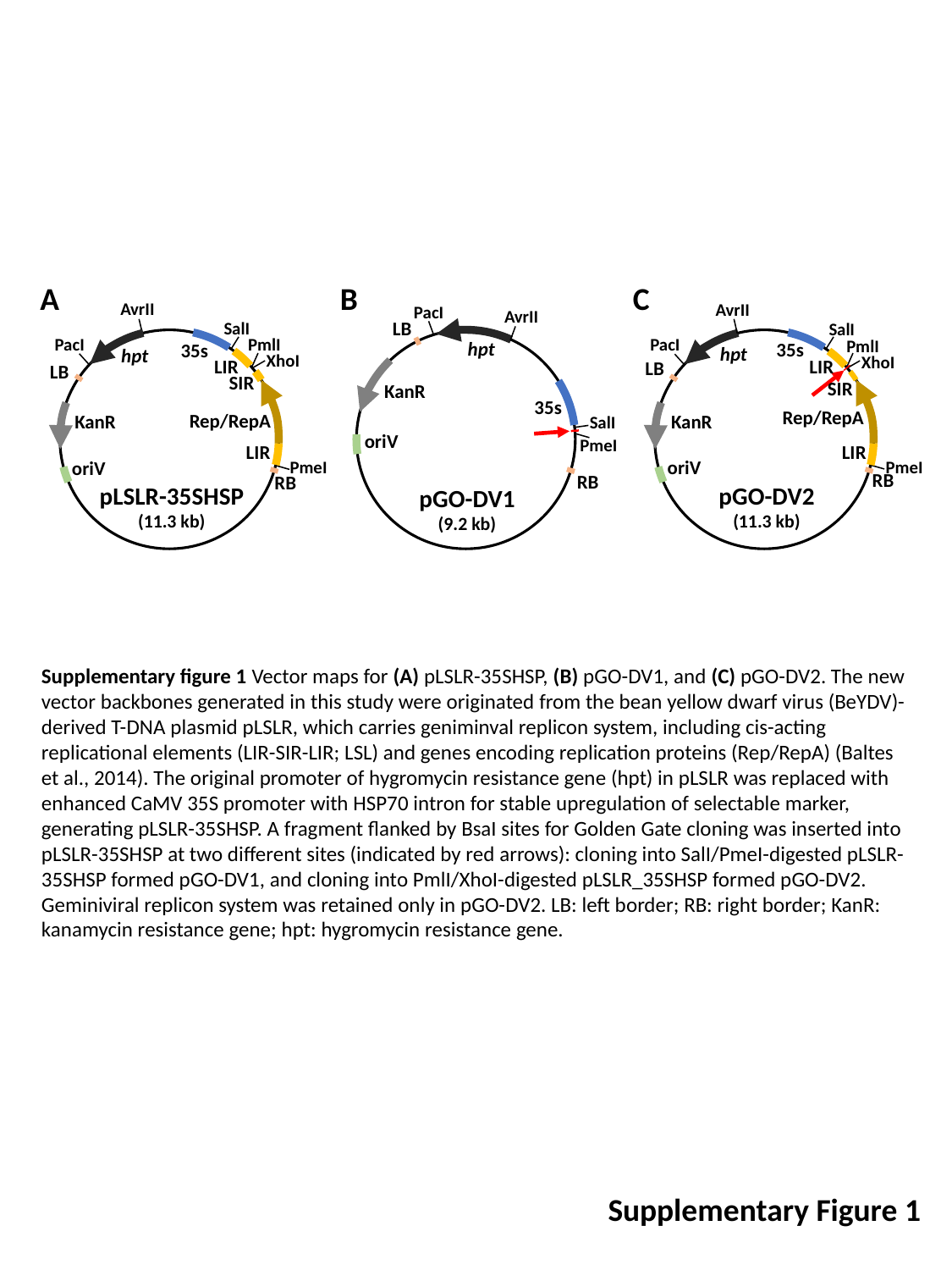

A
B
C
AvrII
SalI
pLSLR-35SHSP
(11.3 kb)
35s
PacI
PmlI
hpt
XhoI
LB
LIR
SIR
Rep/RepA
KanR
LIR
oriV
PmeI
RB
AvrII
SalI
pGO-DV2
(11.3 kb)
35s
PacI
PmlI
hpt
LB
XhoI
LIR
SIR
KanR
Rep/RepA
LIR
oriV
PmeI
RB
PacI
AvrII
LB
pGO-DV1
(9.2 kb)
hpt
KanR
35s
SalI
oriV
PmeI
RB
Supplementary figure 1 Vector maps for (A) pLSLR-35SHSP, (B) pGO-DV1, and (C) pGO-DV2. The new vector backbones generated in this study were originated from the bean yellow dwarf virus (BeYDV)-derived T-DNA plasmid pLSLR, which carries geniminval replicon system, including cis-acting replicational elements (LIR-SIR-LIR; LSL) and genes encoding replication proteins (Rep/RepA) (Baltes et al., 2014). The original promoter of hygromycin resistance gene (hpt) in pLSLR was replaced with enhanced CaMV 35S promoter with HSP70 intron for stable upregulation of selectable marker, generating pLSLR-35SHSP. A fragment flanked by BsaI sites for Golden Gate cloning was inserted into pLSLR-35SHSP at two different sites (indicated by red arrows): cloning into SalI/PmeI-digested pLSLR-35SHSP formed pGO-DV1, and cloning into PmlI/XhoI-digested pLSLR_35SHSP formed pGO-DV2. Geminiviral replicon system was retained only in pGO-DV2. LB: left border; RB: right border; KanR: kanamycin resistance gene; hpt: hygromycin resistance gene.
Supplementary Figure 1

## Slide 2
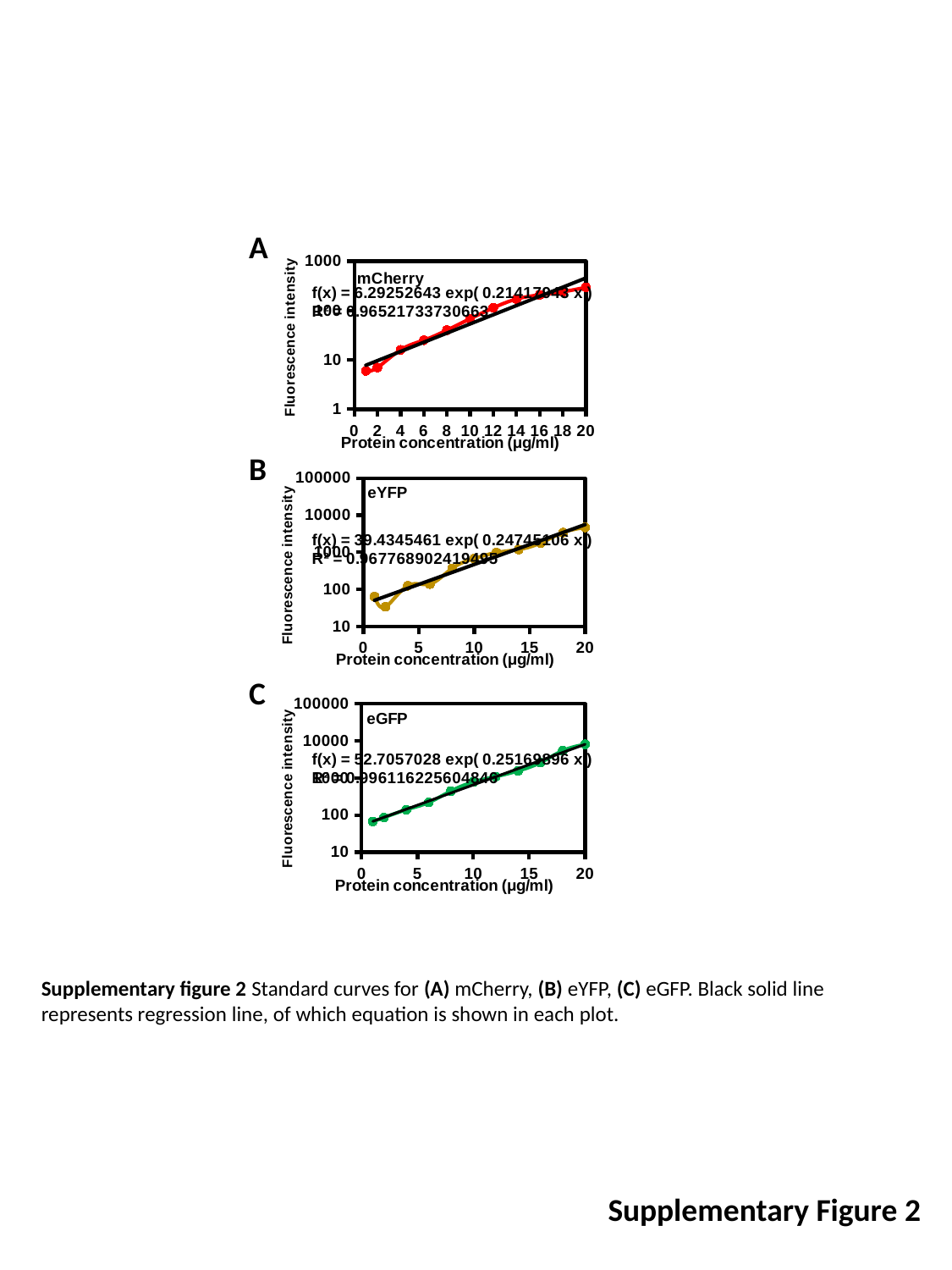

A
### Chart:
| Category | mCherry |
|---|---|
### Chart:
| Category | eYFP |
|---|---|
### Chart:
| Category | eGFP |
|---|---|B
C
Supplementary figure 2 Standard curves for (A) mCherry, (B) eYFP, (C) eGFP. Black solid line represents regression line, of which equation is shown in each plot.
Supplementary Figure 2

## Slide 3
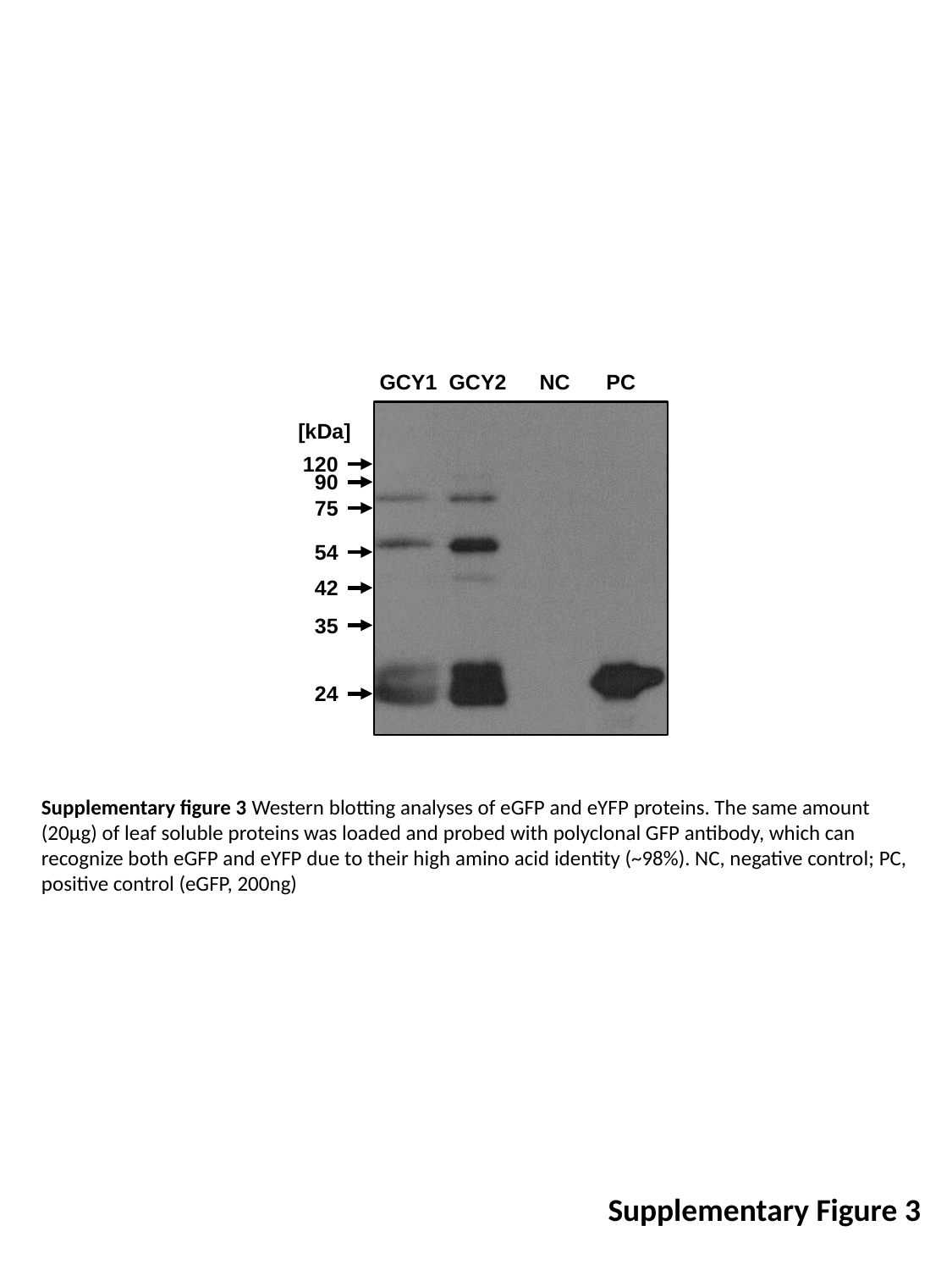

GCY1
GCY2
NC
PC
[kDa]
120
90
75
54
42
35
24
Supplementary figure 3 Western blotting analyses of eGFP and eYFP proteins. The same amount (20μg) of leaf soluble proteins was loaded and probed with polyclonal GFP antibody, which can recognize both eGFP and eYFP due to their high amino acid identity (~98%). NC, negative control; PC, positive control (eGFP, 200ng)
Supplementary Figure 3
