# Supplementary material for: Golden Gate Cloning-Compatible DNA Replicon/2A-Mediated Polycistronic Vectors for Plants
Source: Front Plant Sci. 2020 Oct 21;11:559365. doi: 10.3389/fpls.2020.559365 (PMC7609577; doi:10.3389/fpls.2020.559365)
Supplement: Supplementary file 2 [file Table_1.DOCX]

**Supplementary Table S1. Primers and oligonucleotides used in this study**

| **Primer** | **Sequence (5**′ **- 3**′**)** |
| --- | --- |
| **Fluorescent proteins CDS module preparation^1)^** | |
| FP 1^st^ position-F | ACGGTCTCG**AACC**ATGGTGAGCAAGGGCGAG |
| FP 1^st^ position-R | TGGGTCTCC**CCTC**CTTGTACAGCTCGTCCAT |
| FP 2^nd^ position-F | ACGGTCTCG**CCCT**GTGAGCAAGGGCGAGGAG |
| FP 2^nd^ position-R | TGGGTCTCC**TAGC**CTTGTACAGCTCGTCCAT |
| FP 3^rd^ position-F | ACGGTCTCG**ACCC**GTGAGCAAGGGCGAGGAG |
| FP 3^rd^ position-R | TGGGTCTCC**ATAT**TTACTTGTACAGCTCGTCCAT |
| **pET21a cloning^2)^** |  |
| pET21a-FluP-F | CAGCCATATGATGGTGAGCAAGGGCGAGGA (*NdeI*) |
| pET21a-FluP-R | GATCCTCGAGCTTGTACAGCTCGTCCATGC (*XhoI*) |
| **qRT-PCR** |  |
| L25-rt-F | CCCCTCACCACAGAGTCTGC |
| L25-rt-R | AAGGGTGTTGTTGTCCTCAATCTT |
| mCherry-rt-F | GCCTGGGACATCCTGTCCCC |
| mCherry-rt-R | CCTCGAAGTTCATCACGCGC |

^1)^ Sequences with underline and bold characters indicate *Bsa*I restriction sites and fusion site (4 bp overhangs), respectively.

^2)^ Sequences with underline indicate restriction enzyme sites
